# Fast determination of indoor radon (^222^Rn) concentration using liquid scintillation counting

**DOI:** 10.1007/s10967-017-5226-x

**Published:** 2017-03-18

**Authors:** H. Bem, M. Długosz-Lisiecka, S. Janiak, D. Mazurek, P. Szajerski

**Affiliations:** 1 Higher Vocational State School in Kalisz, ul. Nowy Świat 4, 62-800 Kalisz, Poland; 2 Institute of Applied Radiation Chemistry, Lodz University of Technology, Wroblewskiego 15, 90-924 Lodz, Poland

**Keywords:** Indoor radon, Diurnal variations, Fast measurements, Liquid scintillation

## Abstract

The indoor ^222^Rn radionuclide was directly absorbed in typical 20 ml glass scintillation vials by passing −3 dm^3^ of ambient air through 16 ml of water-immiscible non-volataile scintillation cocktail Ultima-Gold F for 10 min. The activity of radon and its two *α*-emitting daughters: ^218^Po and ^214^Po, was determined with the BetaScout low-background liquid scintillation counter. The limit of ^222^Rn detection is 9 Bq/m^3^, and the quantification limit with 20% relative accuracy is 28 Bq/m^3^. The results of the indoor Rn measurement in different houses showed good consistency with results obtained from a Sarad EQF 3220 device.

## Introduction

 Radon is known to present a risk of lung cancer when it, or rather its decay products, are inhaled [1]. Most of the radon that enters indoor air comes directly from soil and this radionuclide can accumulate there to higher concentrations above 100 Bq/m^3^. For the practical implementation of a radon protection strategy, the International Commission on Radiological Protection (ICRP) recommended an upper value of the so called derived reference level of 300 Bq/m^3^ for ^222^Rn in dwellings [2]. However, on the basis of new epidemiological findings, and consequently the new value of the radon dose conversion coefficient, indoor exposition to 300 Bq/m^3^ corresponds to a higher annual dose within the range of 15–20 mSv in homes [3]. That range of an effective radiation dose exceeds the total average annual exposure of humans from remaining natural radiation sources by at least ten times, equal to −1.2 mSv. The recent evidences on the risks of very low-level radiation exposure seems to support the LNT (linear no-threshold) model and indicates harmful radiation effects well below 100 mSv. In particular, statistically sound epidemiological studies indicate adverse effects to people exposed to very low doses of −10 mSv, e.g., from medical CT (computer tomography) scans on infants [4, 5], to Chernobyl clean-up workers [6], and they even reveal adverse effects from background radiation to which all of us are exposed [7].

Therefore, the Commission strongly encourages national authorities to set a national derived radon reference level as low as reasonably achievable in the range of 100–300 Bq/m^3^ [8]. A similar approach has been proposed in the European Union (EU) commission’s recommendations: under the national action plan, Member States shall promote action to identify dwellings with radon concentrations (as an annual average) exceeding the reference level [9].

A major source of uncertainty in radon risk assessment is the radon dose estimate. Methods for radon exposure measurement in homes are one of the factors that affect the risk estimates in a case–control study. Various methods exist to monitor ^222^Rn in the air. There are several commonly used types of detection, for example: alpha track, activated charcoal adsorption, or instrumental continuous radon monitors. However, there is a need for a fast, relatively simple and cheap method, which can be applied for a preliminary, large scale radon screening in houses with a quantification limit far below 100 Bq/m^3^. The passive diffusion activated charcoal canister PicoRad technique, commonly used for this purpose, requires detector exposition of at least 24 h, an 8-hour period where a scintillation cocktail is added and the radon is allowed to elute, and correction for temperature fluctuations in the examined rooms [10].

Among several procedures described in the literature for direct indoor Rn measurements, liquid scintillation plays a very important role. This technique, utilizing the high solubility of the gaseous Rn in aromatic solvents (common scintillation solvents), was introduced by Horrocks five decades ago [11] and different versions were published until the first decade of this century [12–19]. Very recently, while preparing this paper, another work using mineral oil as a solvent for radon absorption and ^218^Po counting was published, but the proposed range of ^222^Rn determination above 500 Bq/m^3^ makes that method impractical for usual indoor radon measurements [20].

The radon partition coefficients between gaseous phase and typical scintillation solvents—*o*-xylene, toluene and hexane—at 20 °C are equal: 12.75, 13.24 and 16.56, respectively [21, 22]. Therefore, simple air bubbling until the concentration of ^222^Rn in the liquid scintillator reached a state of equilibrium in the ambient temperature is connected with the evaporation of part of the harmful solvent into the environment. The exposition of the open vial without bubbling can only be applied to high soil gas radon concentrations [13, 14] or with a special counter construction with very low background for *α*–particle detection [18, 19]. An alternative method, which depends on 10–20 l of air passing through 20 ml of scintillation solvent held at −78 °C in a bath of dry ice and acetone, gave satisfactory radon activities captured in scintillation vials, but its applications for routine large scale screening purposes are limited [16, 17].

In recent decades, a new class of liquid scintillator cocktails based on diisopropylnaphthalene, with very low vapour pressure and excellent detection efficiency, appeared on the market. In conjunction with a new generation of portable liquid scintillation counters, such a combination allows, for example, for simple radon extraction from 10 ml water samples into 10 ml of water-immiscible scintillation cocktail, directly in 20 ml vials and two-phase counting according to the Prichard and Gessler procedure [23]. Using this scintillator and two phase liquid scintillation counting of *α*-pulses coming from ^222^Rn and two its daughters: ^218^Po and ^214^Po, in the transient radioactive equilibrium, we previously successfully applied that method for low level radon determination in different kinds of groundwater samples [23].

Based on the Cantaloub data, the estimated theoretical value of the gaseous radon partition coefficient between air and the Utima-Gold F diisopropylnaphthalene scintillator at 20 °C should be around 9, slightly lower than that for toluene [20]. The aim of this study was to check the possibility of the direct absorption of indoor radon in this scintillator cocktail at ambient temperatures in 20 ml scintillation vials and to check the obtained quantification limits using a portable scintillation counter with *α*/*β* separation mode.

## Materials and methods

### Theoretical background of the method

#### Theoretical calculation of the calibration coefficient for the proposed method

Assuming a full equilibrium between the concentration of ^222^Rn in gaseous and scintillation phases, one can state:

1 $$K_{\text{H}} = \frac{{C_{\text{SC}} }}{{C_{\text{Rn}} }},$$

where *C*_Rn_ and *C*_SC_ denote concentrations of ^222^Rn in (Bq/m^3^) in air and scintillator phases, respectively. and *K*_H_ -Henry’s constant for scintillation cocktail.

Introducing;

2 $$C_{\text{SC}} = \frac{{I_{\text{SC}} }}{{E \cdot V_{\text{SC}} }},$$

where *I*_sc_- is a measured radon activity in counts per second [cps], *E* is the sum of the detection efficiencies of ^222^Rn, ^218^Po and ^214^Po nuclides (*E* ≤ 3) and *V*_sc_ is the volume of scintillation cocktail [m^3^], *V*_sc_ = 16 × 10^−6^ m^3^.

We obtain:

3 $$C_{\text{Rn}} = \frac{{I_{\text{SC}} }}{{E \cdot V_{\text{SC}} \cdot K_{\text{H}} }},$$

For the constant temperature value of the expression:

4 $$K_{\text{e}} = \frac{1}{{K_{\text{H}} \cdot E \cdot V_{\text{SC}} }}$$

is also constant and it is the so called calibration coefficient—*K*_*e*_. Finally, Eq. (4) can be written:

5 $$C_{\text{Rn}} = K_{\text{e}} \cdot I_{\text{SC}}$$

In the state of equilibrium from the specific activity of the ^222^Rn in scintillation solution, the corresponding indoor radon concentration can be simply calculated after determining the calibration coefficient values by exposure of the vials in the radon chamber with the exactly known ^222^Rn concentration. In practice, an additional term describing the decay of ^222^Rn from absorption to the end of counting must be added, and the final working formula is as follows:

6 $$C_{\text{Rn}} = K_{\text{e}} \cdot I_{\text{SC}} \cdot {\text{e}}^{{0.693 \cdot {{\Delta t} \mathord{\left/ {\vphantom {{\Delta t} {91.8}}} \right. \kern-0pt} {91.8}}}}$$

where Δ*t* is the delay time in (h) between the end of absorption and the end of activity counting.

### Experimental

**Fig. 1:**
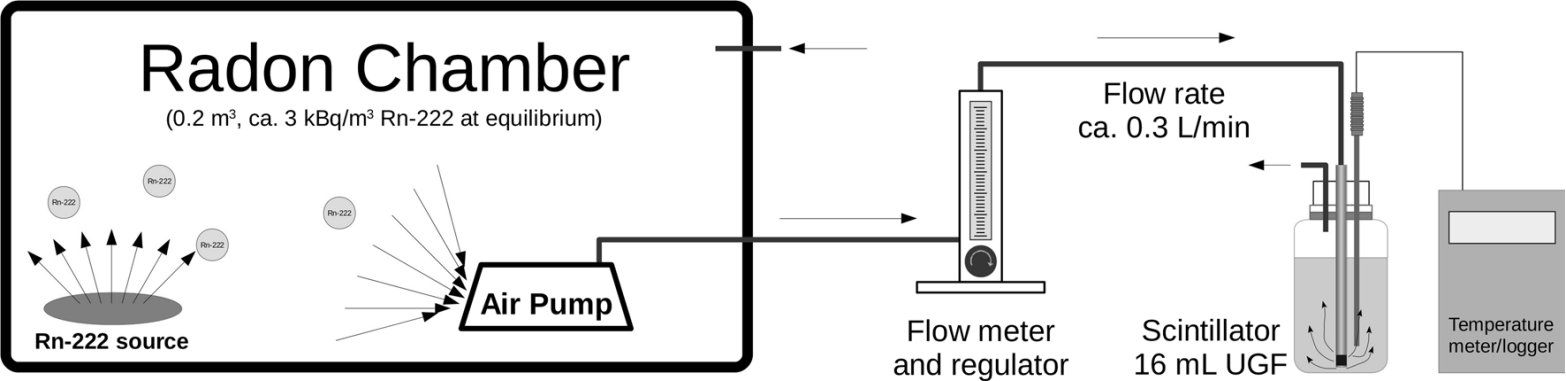
Diagram of radon extraction to the scintillation vials

Ultima-Gold F scintillation cocktail was purchased from PerkinElmer Inc. For radon sampling, 16 ml of the scintillation cocktail was placed in standard 20 ml liquid scintillation vials. In the calibration experiments, the radon from the experimental radon chamber with a volume of 200 dm^3^ was slowly bubbled by means of a small commercial air pump with a volume air flow rate of 0.3 dm^3^/min through a typical G3 Schott type glass filter. After 10 min of bubbling, the Schott filter was removed and the vials with the absorbed radon were immediately closed with a cap. The scheme of the radon sampling is shown in Fig. 1. The concentration of ^222^Rn in the radon chamber was measured by Rad7 device directly before and after a series of calibration experiments. The actual activity values were obtained after sampling time correction, taking into account linear decrease in ^222^Rn concentration in the chamber.

The collected samples were transferred to the laboratory with a delay not exceeding one day. The samples were counted after a minimum 3-h waiting period to ensure the transient radioactive equilibrium between ^222^Rn and its daughters had been reached. The details of *α*-radioactivity counting by the BetaScout (PerkinElmer) device and its quality assurance checking is described elsewhere [24].

Radon fluctuations in two occupied rooms were measured by this method, with 4- to 6-h intervals, and the results were compared with those obtained by the calibrated EQF 3220 (SARAD GmbH) continuous radon monitor.

## Results and discussion

**Fig. 2:**
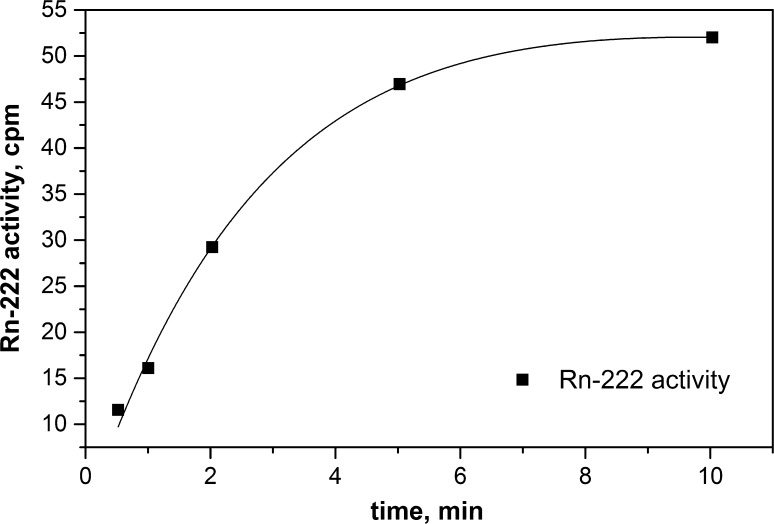
Kinetics of radon absorption in Ultima Gold F scintillator

**Fig. 3:**
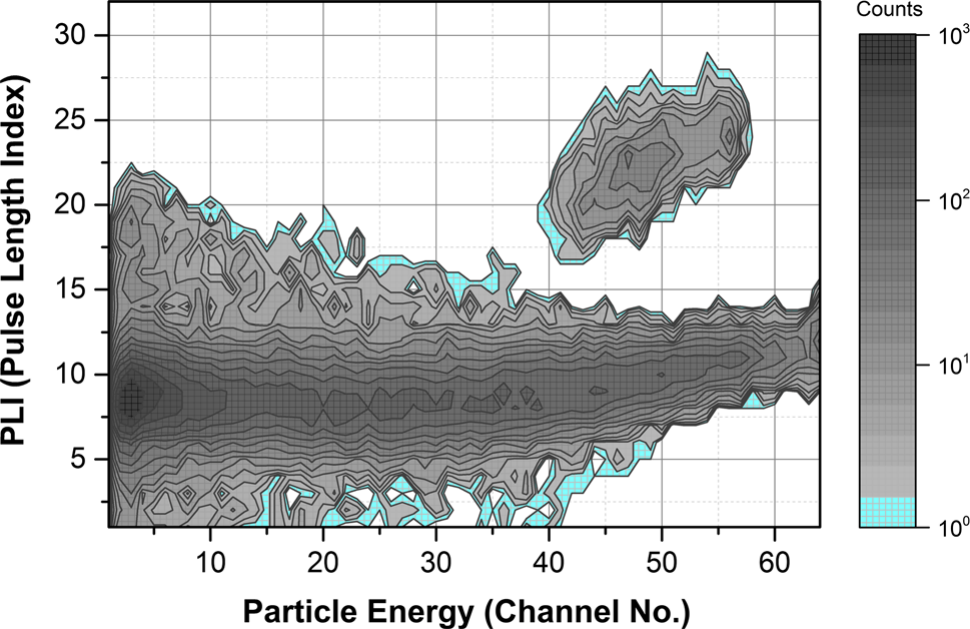
Choice of the region for *α* pulses counting in BetaScout device

Taking into account the value of Henry’s constant *K*_H_ equal to −9 for Ultima Gold F-air system in the ambient temperature, one can simply calculate that the equilibrium radon content for 16 ml of this scintillator is present in around 200 ml of the air volume. However, two-phase mass exchange is quite a complex phenomenon and the radon concentration in the scintillator phase will depend on the flow rate, bubble size and its contact time. Therefore, dependence of the radon activity in solution during bubbling time is a typical saturation curve and for the pumping rate of 0.3 dm^3^ of air per minute is shown in Fig. 2. It means that passing −3 dm^3^ of air (15-fold excess) is sufficient to achieve a state of equilibrium for radon between the two phases. After a delay time of 3 h, a transient radioactive equilibrium is settled in the scintillation vials and three alpha particles can be easy counted in the well separated region (channel no. 40–60 and PLI 16–28) of the BeatScout device (Fig. 3). The precise choice of this region is very important to obtain a very low background: 20 counts for 14,400 s in this counting mode.

According to the van’t Hoff physical absorption model of gases, their solubility in liquids depends on temperature:

7 $$lnK_{\text{H}} = A + \frac{{\Delta H_{\text{S}} }}{\text{RT}}$$

where A—constant, Δ*H*_S_—molar enthalpy of radon solution, J/mol, *T*—temperature, *K*. Substituting (4) into Eq. (6), one can obtain:

8 $$lnK_{\text{e}} = A' - \frac{{\Delta H_{\text{S}} }}{\text{RT}}$$

**Fig. 4:**
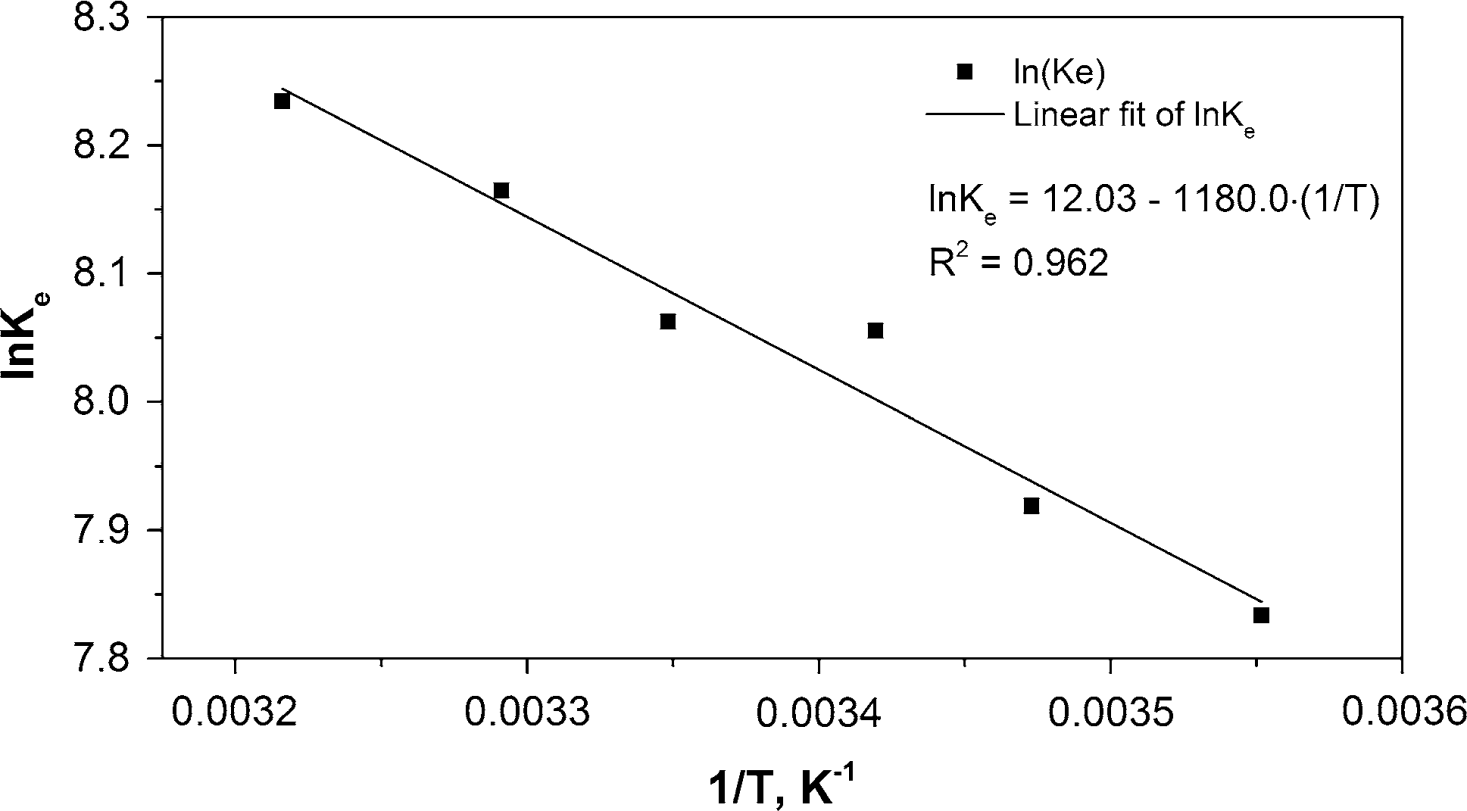
Dependence of the calibration coefficient on (1/T)

Therefore, the same type of relationship should be valid for the dependence of the empirically determined calibration coefficient *K*_e_ and temperature. The relation between ln*K*_e_ and (1*/T*) is shown in Fig. 4.

The obtained values of *K*_e_ for the elaborated method ranged from 2530 for 8 °C to 3770 (Bq/m^3^ cps) for 38 °C. Calculated from the slope of the linear relationship value of the molar enthalpy of radon solubility in Ultima Gold F Δ*H*_s_ = 9.8 kJ/mol is close to values of Δ*H*_*s*_ calculated for the other solvents, hexane and toluene, from the figures presented in Prichard’s paper [16].

### Limits of detection—LD and determining LO_20_ for the method

The calculated values of the calibration coefficients allow us to determine the detection limit of the method using Currie’s expression [25]. For a counting time of 3600 s and background in the *α*-region of the BetaScout device equal to 1.39 × 10^−3^ cps, LD = 9 Bq/m^3^ at 20 °C. For the same assumptions, the limit of determination with 20% relative accuracy, LO_20_ = 28 Bq/m^3^. Therefore, this method allows for fast and quite accurate radon concentration measurements in the majority of dwellings.

#### Application of the method for intercomparison measurements of radon in the Kowary mines

In September last year, a series of intercomparison measurements of the radon concentration in gallery #9 of the abandoned uranium mine in Kowary, Poland, was performed under supervision of the Polish Radon Centre. The results of the one set of experiments with the participation of the liquid scintillation method are shown in Table 1.

**Table 1:** Comparison of Rn-222 concentration determinations by three methods

Sampling	Temp °C	Betascout, I_net_, cps	K_e_ (Bq/m^3^ × cps)	A_sc_, Bq/m^3^	A_RAD7_, Bq/m^3^	A_EQF3020_, Bq/m^3^
Gallery #9	15.0	0.540	2791	1507 ± 49	1410 ± 130	1316 ± 83
Gallery #9	11.8	0.478	2670	1277 ± 44	1190 ± 120	1172 ± 79
Gallery #9	11.2	0.402	2650	1065 ± 40	947 ± 110	815 ± 64

It is evident from the obtained differences in the radon concentrations are basically in the range of accuracy of simultaneous radon measuerements by two calibrated commercial devices: RAD7 and EQF3020. Relative discrepancies of these three methods used do not exceed the tolerable limit of 20%.

#### Examination of the daily variations of indoor radon concentrations in two buildings

Short-term radon concentration measurements are recommended for actual exposure assessment caused by diurnal variations of Rn concentrations in residential buildings. In the Central Europe region, outdoor and indoor radon concentrations are subject to high diurnal variations by a factor of as much as 10 [26] with maximums in the early morning hours [27]. This trend is usually caused by temperature inversion conditions in the ground level air layer, when, during the night, its temperature increases with the altitude up to several metres above the ground. These phenomenon additionally causes increase in negative pressure in the basement resulting in suction of the radon from adjacent soil. This process retards the vertical mixing ratio, and radon escaping from the soil surface is, in the early morning, trapped in the ground level layer and intensively diffuses into the houses. Therefore, the proposed method can be used for fast monitoring of actual radon concentrations over the whole day.

**Fig. 5:**
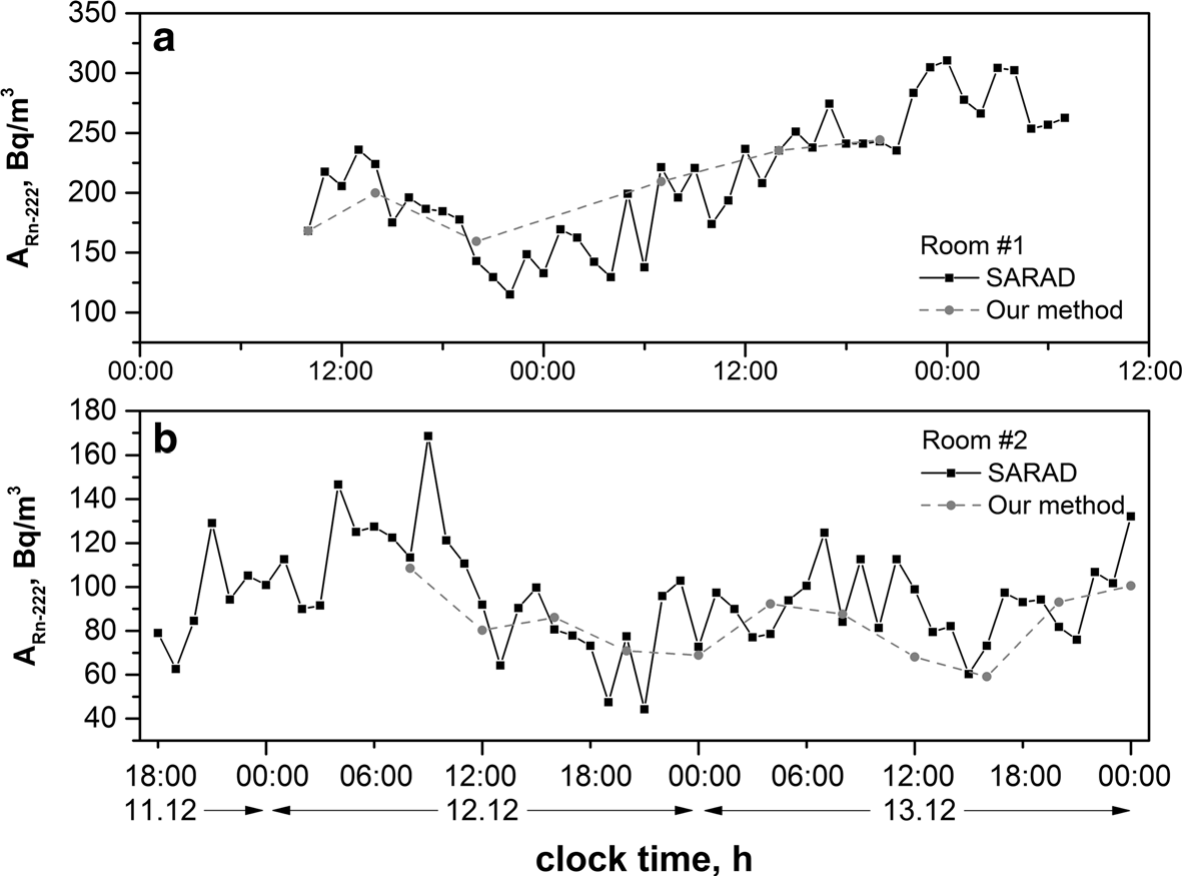
Comparison two methods for radon fluctuation observation in room #1 (*a*) and room #2 (*b*)

A comparison of the radon concentration fluctuations for two different buildings, determined by the Sarad EQF3220 radon monitor and by this method, is shown in Fig. 5a, b.

The first room was located in the corner of a bungalow-type house used as a hostel in Kalisz, a town in Central Poland, and the observed radon concentrations were in the range of 120–320 Bq/m^3^.

Room #2 was situated on the first floor of a two-storey residential building in Lodz. In this room, the radon concentrations fluctuated from a relatively low concentration −40 Bq/m^3^, to a maximal value of 180 Bq/m^3^. It is worth underlining that for both rooms, these two methods show similar values of the radon concentration for selected samples collection times, and the discrepancies between the corresponding results did not exceed 20%. Therefore, the elaborated liquid scintillation method proved its utility, even for low indoor concentrations −50 Bq/m^3^. Moreover, in both rooms, the maximal daily radon concentration occurred in the morning hours, in line with expectations, whereas lower values were observed in the late afternoon hours. On the basis of these and other observations [28, 29] for Polish houses, one can assume that a single measurement of radon concentration at morning may be sufficient for preliminary screening of the expected radon level in the house. These measurements were performed in the winter season, and for the evaluation of the annual doses from indoor radon exposure, similar measurements in spring, summer and autumn should be made.

## Conclusions

The proposed method based on a simple 10–15 min bubbling of indoor air through 16 ml of water-immiscible scintillator with low vapour pressure in conjunction with a portable liquid scintillation counter with *α*/*β* separation option can be applied for large scale screening of indoor radon concentrations. The ^222^Rn nuclide quantification level LO_20_ of this method equal to 28 Bq/m^3^ makes this method extremely convenient for the preliminary selection of buildings with indoor concentrations exceeding the value of 100 Bq/m^3^ i.e., the expected radon reference levels accepted by the majority of EU countries. The method is very cheap and environmentally friendly, as the scintillation solution can be reused after a one-month.
